# Fabrication and spectroscopic investigation of branched silver nanowires and nanomeshworks

**DOI:** 10.1186/1556-276X-7-596

**Published:** 2012-10-27

**Authors:** Xiao-Yang Zhang, Tong Zhang, Sheng-Qing Zhu, Long-De Wang, Xuefeng Liu, Qi-Long Wang, Yuan-Jun Song

**Affiliations:** 1 School of Electronic Science and Engineering, Southeast University, Nanjing 210096, People's Republic of China; 2 Key Laboratory of Micro-Inertial Instrument and Advanced Navigation Technology, Ministry of Education, Nanjing 210096, People's Republic of China; 3 Suzhou Key Laboratory of Metal Nano-Optoelectronic Technology, Suzhou Research Institute of Southeast University, Suzhou, 215123, People's Republic of China; 4 Institute of Optics and Electronics, CAS, PO Box 350, Shuangliu, Chengdu, 610209, China

**Keywords:** Silver Nanowires, Nanomeshworks, Branched nanostructures, Localized surface plasmon resonance, Hot spots, Bandwidth

## Abstract

Wide wavelength ranges of light localization and scattering characteristics can be attributed to shape-dependent longitude surface plasmon resonance in complicated nanostructures. We have studied this phenomenon by spectroscopic measurement and a three-dimensional numerical simulation, for the first time, on the high-density branched silver nanowires and nanomeshworks at room temperature. These nanostructures were fabricated with simple light-induced colloidal method. In the range from the visible to the near-infrared wavelengths, light has been found effectively trapped in those trapping sites which were randomly distributed at the corners, the branches, and the junctions of the nanostructures in those nanostructures in three dimensions. The broadened bandwidth electromagnetic field enhancement property makes these branched nanostructures useful in optical processing and photovoltaic applications.

## Background

Noble metal nanostructures supporting surface plasmons arising from the coherent oscillations of the conduction electrons under the radiation of the incident light have been widely investigated [1-24]. The optical spectral signatures of the plasmonic nanostructures are mainly dependent on the distribution of the electromagnetic field on the surface of the metal nanostructures. When the size of the metal nanostructures is smaller than the wavelength of the incident light, they support localized surface plasmon resonance (LSPR) at particular resonance wavelengths [1]. The LSPR properties of the plasmonic nanostructures are size and shape dependent [1-3]. With the increase of the size of the metal nanostructures, high-order resonance occurs owing to the surface plasmon polariton (SPP) mode interferences. For example, metal nanowires support the Fabry-Perot (FP) modes [6], micron-size metal nanoplates support the whispering gallery modes [7], and the flag type of metal nanostructure supports competitive LSPR and FP modes simultaneously [23]. These metal nanostructures with regular shapes usually exhibit narrow-band resonance characteristic seen from their extinction spectra.

As well known, on the contrary to those elemental nanoparticles, metal nanostructures with complicated geometries, such as nanochains and branched nanowires assembled or fused by the elemental nanoparticles, show enhanced light-trapping ability [8-24]. When the metal nanoparticles are in close proximity or jointed together, the intensity of the localized electromagnetic field at the corner area and the junctions of these nanostructures are dramatically enhanced owing to the existing plasmon coupling between different propagation modes or resonance modes [8,9]. It is usually accompanied by obvious redshift and extension of their resonance bands [3,9]. The overall optical properties including obvious light-trapping and scattering enhancement in a wide wavelength range make these complicated metal nanostructures highly desirable in improving the performance of bio-chemical sensors [21,22], solar cells [24,25], and wide-bandwidth light-emitting diodes [26]. Although considerable progress has been made in the fabrication methods of such complicated metal nanostructures, such as laser welding [8-10], sintering triggered thermally [11] or chemically [12], electrodeposition [13], microwave irradiation [14], and chemical synthesis methods [15-22], it is still a challenge to fabricate high-yield metal nanostructures with excellent light-trapping property in a wide wavelength range to meet the practical demands for a large-scale production.

In this study, we present a simple and facile light-induced colloidal method to fabricate high-density branched silver nanowires and nanomeshworks. These complicated nanostructures showed significant light-trapping and scattering properties in the wide range from the visible to the near-infrared wavelengths, arising from the shape-dependent longitude surface plasmon resonance. The broadened bandwidth electromagnetic field enhancement characteristic makes these nanostructures useful in optical processing and photovoltaic applications.

## Methods

The fabrication process of the branched silver nanowires and nanomeshworks includes two steps.

### Synthesis of the silver seeds

The first step is the synthesis of the silver seeds. A 5-mL amount of 0.5 M trisodium citrate, 0.5 mL of 0.05 M l-arginine, 0.15 mL of 0.5 M polyvinylpyrolidone (PVP), and 0.2 mL of 0.5 M silver nitrite were added to 7 mL of deionized water while stirring slowly. Then, 2 mL of 0.4 M sodium borohydride (NaBH_4_) was added to the solution slowly drop by drop. The color of the silver seed solution changed to dark brown rapidly.

### Fabrication of the branched silver nanowires and nanomeshworks

**Figure 1:**
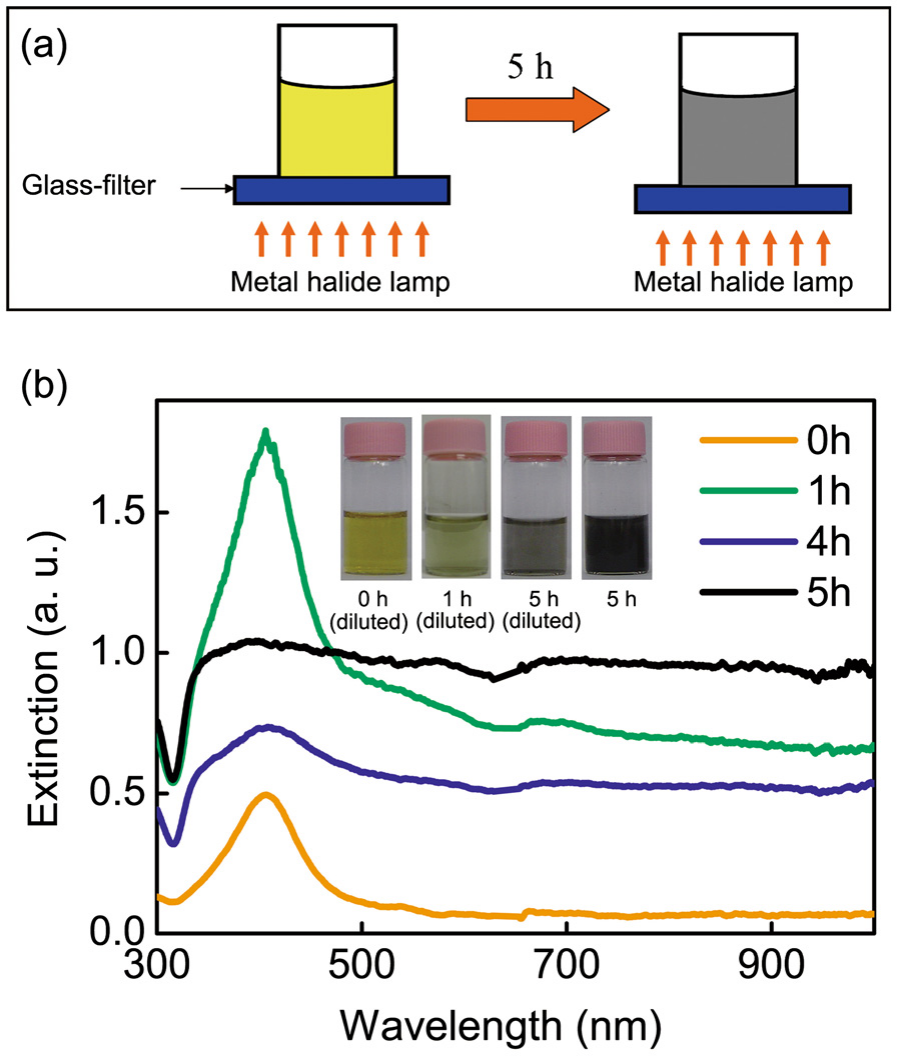
**Scheme of the experiment and the optical characteristics of the silver solution. **(**a**) The exposure process for the fabrication of branched silver nanowires and nanomeshworks. (**b**) The extinction spectra and the photographs of the silver nanostructure solutions at different exposure times.

The silver seed solution was stirred slowly for 20 min in a dark place. Then, it was exposed to a 400-W metal halide lamp covered by a blue glass filter (B-440 UV–vis bandpass filter, Edmund Optics Inc., Barrington, NJ, USA). The strategy of the light exposure procedure is shown in Figure 1a. A beaker containing silver seed solution was placed on the top surface of the glass filter. The top and the side wall of the beaker were covered by aluminum foils to avoid the influence of ambient light. The metal halide lamp was placed below the glass filter. The density of the light intensity passing through the glass filter is approximately 0.5 mW/cm^3^. It can be varied by tuning the distance between the lamp and the glass filter. It is necessary to shake up the solution for several times to avoid the aggregation of the nanostructures during the exposure procedure. When the silver seeds were exposed to the blue light for approximately 5 h, high density branched nanostructure solution with black color were achieved. Considering H_2_ as an outgrowth when NaBH_4_ is used as a reducing agent in the reduction procedure, it is necessary to keep aeration when the solution with such a high material concentration is exposed to the light.

## Results and discussion

**Figure 2:**
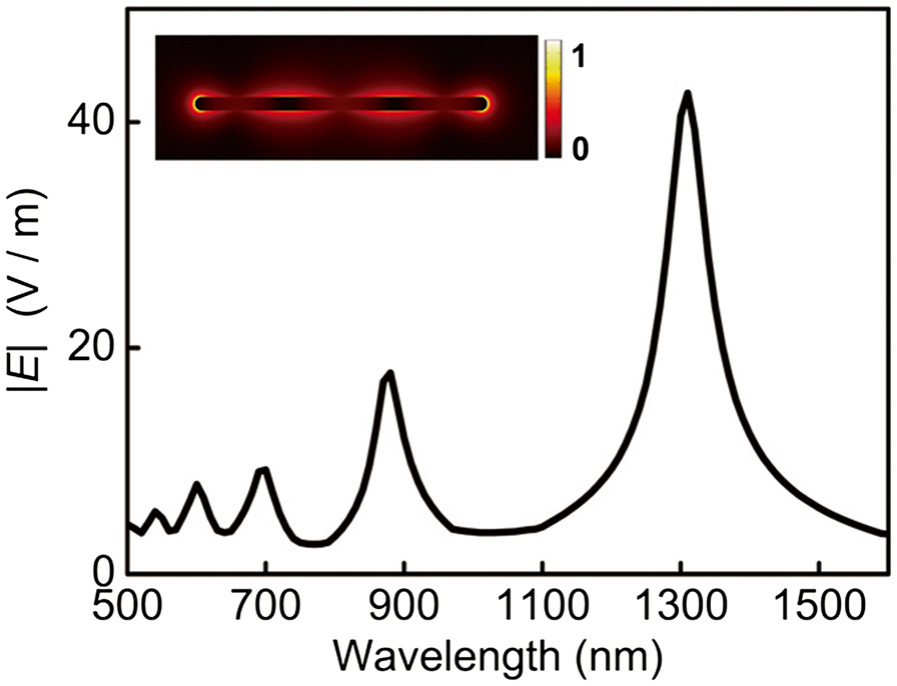
**LSPR spectrum. **Bare silver nanowire with a diameter of 30 nm and a length of 600 nm. The nanowire is surrounded by water (n = 1.33). The inset shows the normalized electric field distribution |E| at the center of the nanowire at a resonance wavelength λ = 1,320 nm.

The color of conventional chemical synthesized silver nanowire solution is pale green accompanied with strong reflection [4] due to the narrow resonance bands and selective light localization properties. Figure 2 shows the typical LSPR spectrum of silver nanowire with smooth surface calculated by three-dimensional finite element method (FEM) [9,27,28]. The silver nanowire supports longitude SPP modes which reflect between its two terminal facets. Therefore, it shows periodic plasmon resonance bands with narrow bandwidth arising from the Fabry-Perot interference of the SPP modes. From the inset in Figure 2, one can see that the electric field is distributed at the surface and the ends of the nanowire. However, light localization and scattering enhancement mainly occur at the ends of the nanowire. Therefore, scattering is not obvious when the SPP modes are transmitted along the nanowires with smooth surface.

Compared with the conventional silver nanowire solution, silver solution prepared in our way containing branched nanostructures shows greatly enhanced light-trapping and scattering characteristics in a wide wavelength range. The two-step light-induced chimerical colloidal method used here was first reported by Pietrobon and Kitaev for the synthesis of decahedral silver nanoparticles [2]. The scheme of the light exposure process is shown in Figure 1a. Surprisingly, silver nanostructures with distinct morphology and optical spectral signatures were found when we increased the material concentration by 100 times higher. Figure 1b shows the extinction spectra of the silver solution measured by a fiber-optic spectrometer (PG2000, Ideaoptics Technology Ltd., Shanghai, China) and the photographs of the solution samples at different exposure times. The first step is the synthesis of the silver seed solution. As shown in the inset of Figure 1b, the color of the diluted silver seed solution (exposure time is 0 h) is light yellow. As shown in the extinction spectrum, a narrow resonance band at 420 nm corresponding to the LSPR wavelength of typical silver nanospheres with a diameter of several nanometers [3] can be observed. The second step is the light-induced regrowth process of the silver nanostructures which is highly dependent on the material concentration. At a low concentration as reported in [2], decahedral silver nanoparticles with uniform sizes and shapes are the ultimate product. However, when the material concentration was increased by 100 times, the ultimate products containing mainly the branched nanowires and nanomeshworks can be observed. As seen from the extinction spectra, the resonance band at 420 nm decreased, and the absorption ratio in the longer wavelength increased obviously with the increase of the exposure time. As the welded metal nanobranches and nanomeshworks increased, the color of the solution darkened rapidly with the extension of the exposure time, which is shown in the inset of Figure 1b. After a 5-h exposure procedure, the color of the solution turned to black which is obviously different from silver nanoparticles with regular shapes, such as bared nanowires [4] or nanoplates [3]. The optical spectra and the photographs of the solution are strong evidences to show that the ultimate silver nanostructures can trap light effectively in a broad wavelength range from the visible to the near-infrared wavelengths.

**Figure 3:**
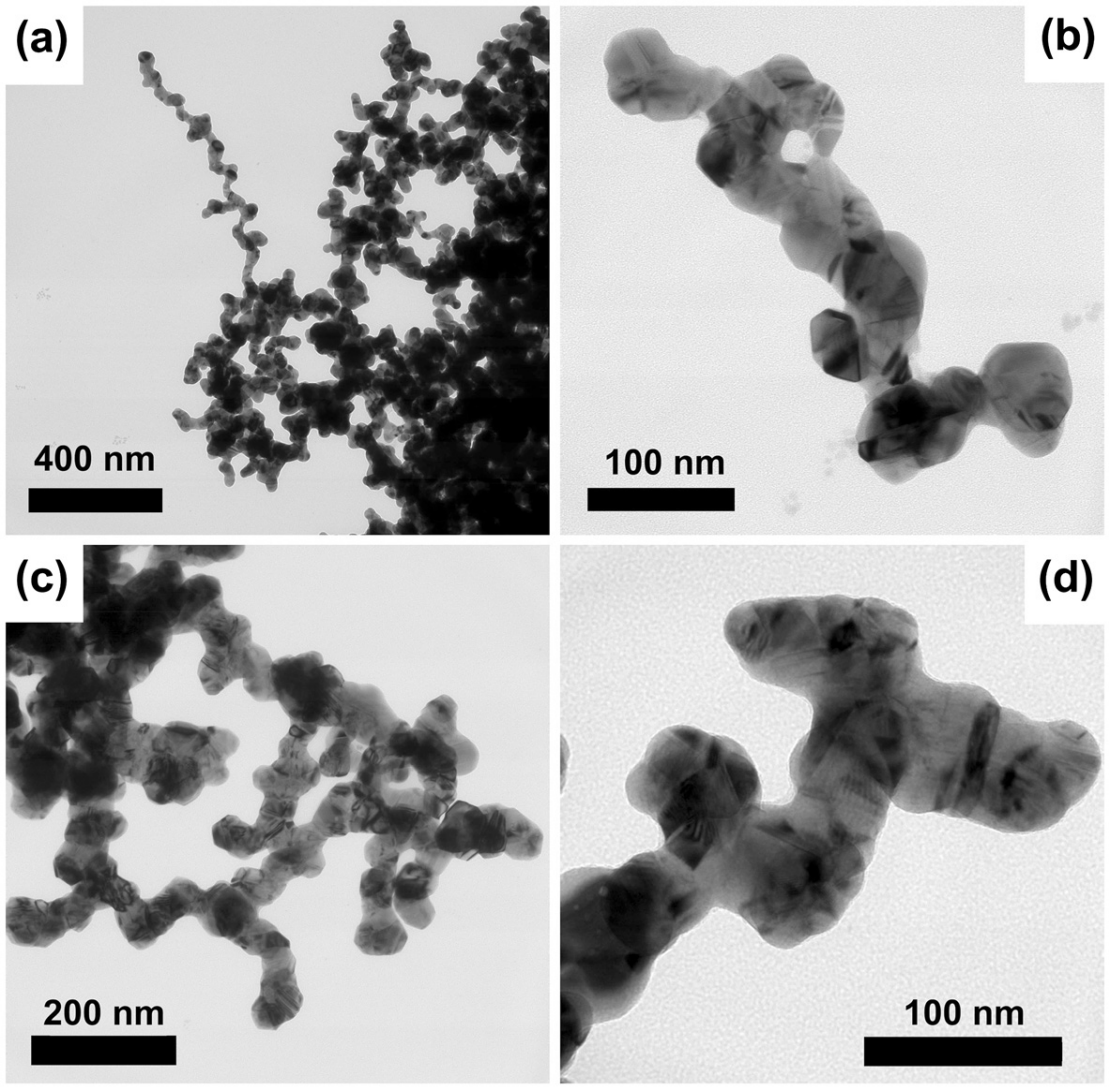
**TEM images of the silver nanowires and nanomeshworks. **(**a**) and (**b**) Typical branched silver nanowires. (**c**) Silver nanomeshworks. (**d**) High-resolution image of the nanomeshwork.

In the light exposure process, the small silver seeds aggregated and welded together when the solution was under exposure. The final morphology of the nanostructures depends on the concentrations of the capping agents, PVP, and l-arginine. As discussed in [18], high PVP concentration serves as a structure-directing agent to keep the nanostructure growth linear. High l-arginine concentration controls the reaction velocity and avoids the size growth of the nanoparticles [2]. Figure 3 shows the transmission electron microscopy (TEM) (Tecnai G2, FEI, Hillsboro, OR, USA) images of the ultimate silver nanostructures after exposure. The TEM samples were washed by ethanol and deionized water and then dispersed uniformly by ultrasonic before measurement. The typical nanostructures include branched nanowires and nanomeshworks. The diameter of these branched nanowires is about several tens of nanometers. Figure 3a,b shows linear branched nanowires with rough surfaces. It was found that there are many knobs and branches in the nanowires. Figure 3c shows nanomeshworks containing many short nanowires connected together randomly in three dimensions. From the high-resolution TEM image in Figure 3d, one can see that the branches have joined together permanently, obviously different from the nanochains assembled by adjacent nanoparticles [17,19,20].

To investigate the light-trapping property of these nanostructures, we use three-dimensional FEM to simulate the electric field distribution of the branched nanostructures. Rectangular wave incoming from the upper *xy* plane was set as the source boundary. The other outer boundaries were set as the scattering boundary condition. The cladding layer surrounding the silver nanostructures is water with a refractive index *n* = 1.33. The dispersion coefficient of water is neglected in the simulation. The dielectric constant of silver is taken from [29].

**Figure 4:**
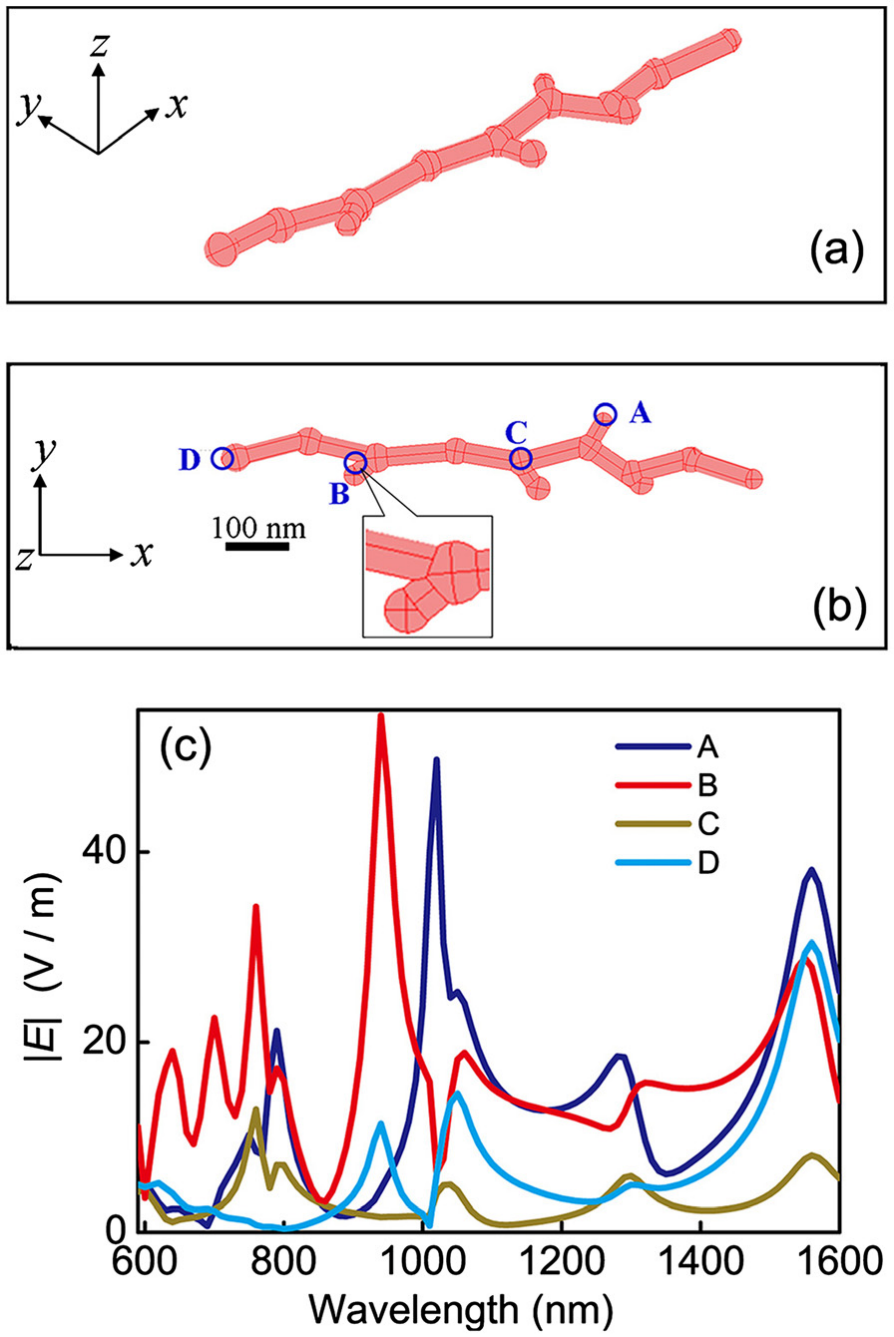
**Simulation of the LSPR spectra at different positions distributed in a branched silver nanowire. **(**a**) and (**b**) The geometry of the silver nanowire from the lateral view and the plane view, respectively. (**c**) The LSPR spectra at different positions highlighted in (**b**).

**Figure 5:**
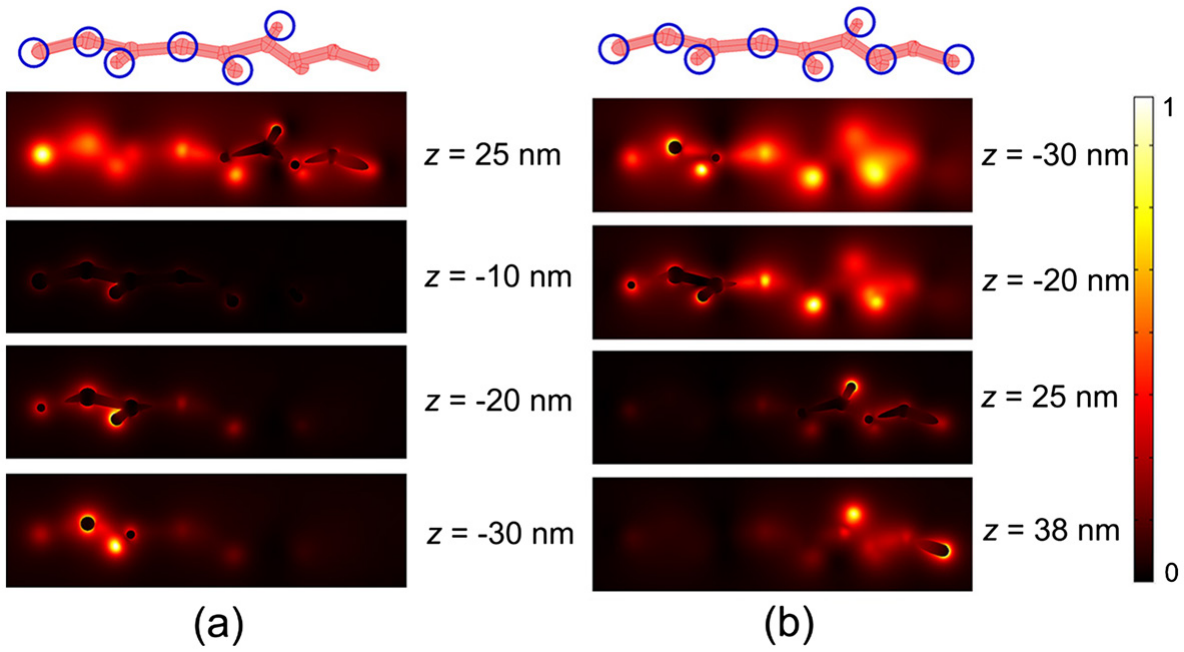
**Simulation of the normalized electric-field |*E*| distributions. **In the branched silver nanowire at different planes along the *z*-axis. The wavelength of the incident light is 940 and 1,020 nm in (**a**) and (**b**), respectively. The blue circles in the geometry images of the nanostructures illustrate the hot spots.

We first investigated the optical property of the branched silver nanowires. In the numerical model, we created a branched nanowire with randomly distributed branches and knobs referred to the morphology shown in the TEM images of Figure 3a,b. The configuration of the silver nanowire is shown in Figure 4a,b. The morphologic characteristics are listed as follows: the nanowire is knotty with an average diameter of 30 nm, the distance between the adjacent knobs is approximately 100 nm, the diameter of the knobs is between 30 and 50 nm, the branches connect to the bus nanowire with random angles, and the total length of the nanowire is approximately 800 nm. Figure 4c shows the relationship between the amplitude of the normalized electric field |*E*| and the wavelength at different positions of the branched silver nanowire including the branch, the corner between two branches, the junction, and the end as labeled by A, B, C, and D, respectively, in Figure 4b. There are many resonance bands covering the wavelength range from the visible to the near-infrared wavelengths in the optical spectra. The bandwidths of the resonance bands are wide, and the amplitudes at the resonance peaks are high, especially at the branches and the corner area. Figure 5 shows the simulation results of the normalized electric field spatial distribution at different *z* positions seen from the *xy* plane. The wavelength of the incident light is 940 and 1,020 nm in Figure 5a,b, respectively. Light can be easily localized at the positions of the knobs and the corners between the branches when it propagated along the nanowires with rough surfaces. It leads to various wavelength-dependent ‘hot spots’ [3,21] which are randomly distributed in the nanostructures. The light intensity at the area of the hot spots can be enhanced greatly, leading to a strong interaction of light with matters.

**Figure 6:**
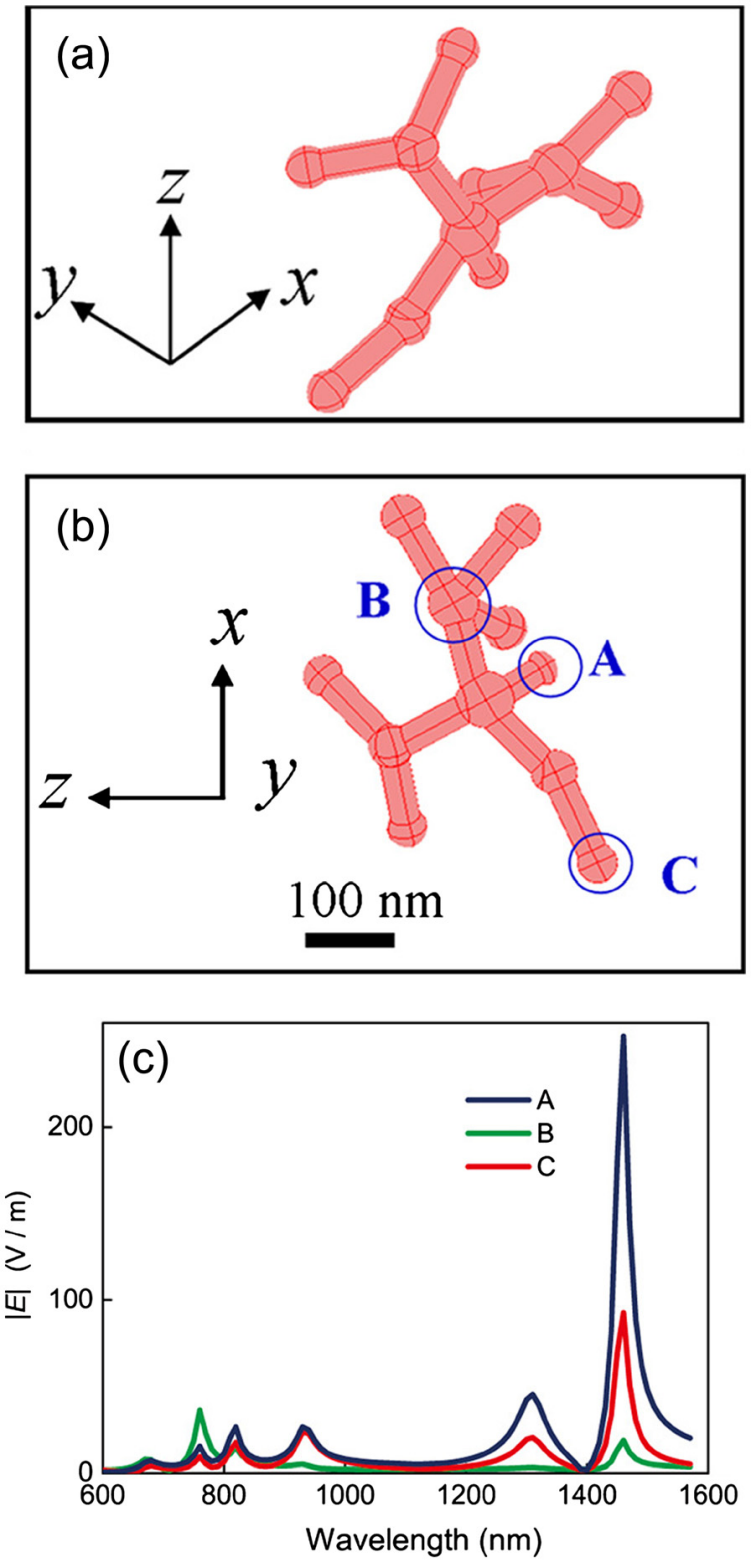
**Simulation of the LSPR spectra at different positions distributed in a silver nanomeshwork. **(**a**) and (**b**) The geometry of the silver nanomeshwork from the lateral view and the plane view, respectively. (**c**) The LSPR spectra at different positions highlighted in (**b**).

**Figure 7:**
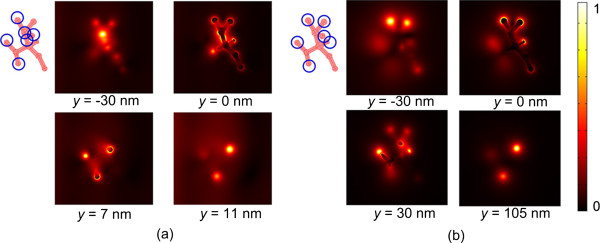
**Simulation of the normalized electric-field |*E*| distributions. **In the silver nanomeshwork at different planes along the *z*-axis. The wavelength of the incident light is 760 and 1,460 nm in (**a**) and (**b**), respectively. The blue circles in the geometry images of the nanostructures illustrate the hot spots.

Figure 6 shows the LSPR spectra at different positions distributed in a silver nanomeshwork. The complicated meshwork is formed by randomly distributed short branches in three-dimensional space, as illustrated in Figure 6a,b. The diameter and the length of the short branches were set to be approximately 30 and 100 nm, respectively. In Figure 6c, the three curves represent the normalized electric field |*E*| at the junction, the branch, and the end of the meshwork highlighted in Figure 6b. The LSPR spectra also exhibit multiple resonance bands with intense light enhancement in a wide wavelength range at the branch and the end area of the nanomeshwork, which is the same phenomenon as the nanowires. However, the light enhancement at the junction area is not obvious. It is because the longitudal resonant cavity length of the nanomeshwork is much shorter than that of the nanowire in our models. For such nanostructures with short resonant cavity length, light is easily be localized at the branches and the corner area rather than the middle area without sharp corners. Figure 7 shows the simulation results of the wavelength-dependent normalized electric field |*E*| distributions in the silver nanomeshwork at different planes along the *z*-axis. All images shown so far indicate that the silver nanomeshwork behaves like a three-dimentional ‘light cage’ which traps light at those randomly distributed branches and knobs. The distribution of the hot spots is also wavelength dependent. Such nanostructures can trap light with different wavelengths effectively in large-space areas.

The above simulation results indicate that such randomly constructed silver nanostructures with rough surfaces and multi-branches have high light-trapping efficiency, in accordance with the measured extinction spectra.

## Conclusions

In this study, we introduce a simple chemical method for the fabrication of high-density branched silver nanostructures. Both experimental measurement and relative three-dimensional numerical simulation results show that these nanostructures have significant properties as follows: First, these branched silver nanostructures have significant light-trapping and scattering properties in a broad wavelength range. Second, light can propagate in a longer distance along the waveguide-like plasmonic nanostructures to improve the interaction between the surrounding materials and light. Third, the fabrication routes with easy operation and high yields meet the satisfaction of the large-scale production. These properties make such nanostructures useful in practice applications, such as photovoltaics, bio-chemical sensing, and optical processing.

## Competing interests

The authors declare that they have no competing interests.

## Authors' contributions

XYZ carried out the main part of synthetic, analytical, and simulation works and drafted the manuscript. SQZ, LDW, and YJS participated in the synthetic and analytical works. TZ, XFL, and QLW participated in the discussion of the experimental details and participated in the draft preparation. All authors read and approved the final manuscript.
